# Beta/Gamma Oscillations and Event-Related Potentials Indicate Aberrant Multisensory Processing in Schizophrenia

**DOI:** 10.3389/fpsyg.2016.01896

**Published:** 2016-12-06

**Authors:** Johanna Balz, Yadira Roa Romero, Julian Keil, Martin Krebber, Michael Niedeggen, Jürgen Gallinat, Daniel Senkowski

**Affiliations:** ^1^Department of Psychiatry and Psychotherapy, St. Hedwig Hospital, Charité – Universitätsmedizin BerlinBerlin, Germany; ^2^Department of Education and Psychology, Freie Universität BerlinBerlin, Germany; ^3^Department of Psychiatry and Psychotherapy, University Medical Center Hamburg-EppendorfHamburg, Germany

**Keywords:** crossmodal, sound-induced flash illusion, oscillatory activity, electroencephalography, perception, attention, audiovisual, neural oscillations

## Abstract

Recent behavioral and neuroimaging studies have suggested multisensory processing deficits in patients with schizophrenia (SCZ). Thus far, the neural mechanisms underlying these deficits are not well understood. Previous studies with unisensory stimulation have shown altered neural oscillations in SCZ. As such, altered oscillations could contribute to aberrant multisensory processing in this patient group. To test this assumption, we conducted an electroencephalography (EEG) study in 15 SCZ and 15 control participants in whom we examined neural oscillations and event-related potentials (ERPs) in the sound-induced flash illusion (SIFI). In the SIFI multiple auditory stimuli that are presented alongside a single visual stimulus can induce the illusory percept of multiple visual stimuli. In SCZ and control participants we compared ERPs and neural oscillations between trials that induced an illusion and trials that did not induce an illusion. On the behavioral level, SCZ (55.7%) and control participants (55.4%) did not significantly differ in illusion rates. The analysis of ERPs revealed diminished amplitudes and altered multisensory processing in SCZ compared to controls around 135 ms after stimulus onset. Moreover, the analysis of neural oscillations revealed altered 25–35 Hz power after 100 to 150 ms over occipital scalp for SCZ compared to controls. Our findings extend previous observations of aberrant neural oscillations in unisensory perception paradigms. They suggest that altered ERPs and altered occipital beta/gamma band power reflect aberrant multisensory processing in SCZ.

## Introduction

An increasing body of literature suggests that individuals with schizophrenia (SCZ) have deficits in the processing and perception of sensory information (Giersch et al., 2013; Onitsuka et al., 2013; Javitt and Freedman, 2015). Research has stated that primary cognitive disturbances in SCZ cause an inability to sufficiently filter and process sensory information, leading to disconnection of information and disrupted binding (McGhie and Chapman, 1961; Shakow, 1963; Freedman et al., 1991; Vlcek et al., 2014). Furthermore, electrophysiological studies have suggested that aberrant neural oscillations play a role for cognitive deficits in SCZ (Andreasen, 2000; Lee et al., 2003; Lisman, 2012; Pittman-Polletta et al., 2015; Senkowski and Gallinat, 2015; Uhlhaas and Singer, 2015; Keil et al., 2016). Thus far, sensory processing deficits have been most consistently observed in studies using setups with unisensory stimulation. For instance, one study found impairments in visual temporal order judgments in SCZ (Capa et al., 2014). The study indicated an extended visual simultaneity threshold in patients compared to healthy control participants (HC). Another study investigated perceptual fusion of visual stimuli using the three-flash illusion paradigm (Norton et al., 2008; Chen et al., 2014). In this paradigm two flashes are perceived either as one, two, or three flashes, depending on the inter-stimulus interval between the two visual stimuli. The study by Norton et al. (2008) revealed that the three-flash illusion occurred more often in SCZ than in HC when the inter-stimulus intervals were longer. When the inter-stimulus interval was shorter, HC reported more illusionary three flashes than SCZ. Thus, there is substantial evidence suggesting altered unisensory stimulus processing in SCZ.

In addition to findings of unisensory processing deficits, there is also some evidence for aberrant multisensory processing in SCZ (Foucher et al., 2007; Stekelenburg et al., 2013; Roa Romero et al., 2016a,b; Stevenson et al., 2016; for a review see Tseng et al., 2015). One study examined simultaneity judgments in unisensory and multisensory stimuli and found that SCZ have a lengthened window of simultaneity for unisensory visual, unisensory auditory, and bisensory audiovisual stimuli (Foucher et al., 2007). Another study investigated multisensory processing of audiovisual video clips in which the onset of the sound was either congruent or incongruent with the visual input (Stekelenburg et al., 2013). Using event-related potentials (ERPs), Stekelenburg et al. (2013) found that SCZ lacked on a reduction of the N1 component to bisensory compared to unisensory auditory stimuli. Such a reduction, which presumably relates to the fact that the visual input precedes the auditory onset, was observed in healthy individuals. The N1 reduction for auditory stimuli is usually interpreted in terms of a valid crossmodal prediction, e.g., when a visual syllable matches an auditory syllable (van Wassenhove et al., 2005). Thus, the absence of the N1 reduction in bisensory stimulation likely reflects aberrant audiovisual processing in patients. Notably, recent studies have also provided first evidence for altered neural oscillations during multisensory processing in patients (Stone et al., 2014; Roa Romero et al., 2016b). Overall, these findings indicate altered multisensory processing in SCZ.

An established paradigm for examining integrative audiovisual processing is the sound-induced flash illusion (SIFI; Shams et al., 2000). Herein, a single flash that is presented alongside two rapidly repeating tones is either perceived as one flash (i.e., no-illusion) or two flashes (i.e., illusion). Hence, this paradigm allows for the direct comparison of physically identical audiovisual stimuli that are either not integrated (i.e., no-illusion) or integrated (i.e., illusion). Thus far, no electrophysiological study has investigated the SIFI in SCZ. However, a number of electroencephalography (EEG) and magnetoencephalography (MEG) studies have examined the neural correlates underlying the SIFI in healthy participants (Mishra et al., 2007; Keil A. et al., 2014; Balz et al., 2016). For instance, in one study by Mishra et al. (2007), ERPs and neural oscillations to SIFI stimuli that either induced or did not induce an illusion were compared. The authors calculated the difference between ERPs of both conditions and revealed an early modulation of activity in the visual cortex about 30–60 ms after the offset of the second auditory stimulus. Subjects with higher illusion rates showed larger amplitudes, which the authors interpreted as individual differences in the neural connectivity underlying multisensory integration. The authors also observed differences between illusion and no-illusion trials that started around 90 ms, i.e., in the time range of the auditory N1 and the visual P1, after the onset of the first tone. Moreover, in agreement with other reports (Bhattacharya et al., 2002), the analyses of neural oscillations revealed power differences between illusion and no-illusion trials in neural oscillations ranging from 25 to 35 Hz, comprising an enhancement of beta/gamma power for illusion compared to no-illusion trials. Notably, previous unisensory studies in SCZ have shown dysfunctional oscillatory activity in this frequency range (Gallinat et al., 2004; Uhlhaas et al., 2006; Spencer et al., 2008; Leicht et al., 2010, 2015). Nevertheless, while the neural signatures underlying the SIFI have been well characterized in healthy individuals, it is unknown whether SCZ show alterations in the perception and processing of the SIFI. In the present EEG study we examined perception, ERPs, and neural oscillations during the SIFI in SCZ and HC. In accordance with recent findings of altered multisensory processing, we predicted that SCZ show alterations in the perception and neural processing of the SIFI.

## Materials and Methods

### Participants

Twenty-four patients with the DSM-IV diagnosis SCZ were recruited from outpatient units of the Charité – Universitätsmedizin Berlin. In addition, 24 age, education, gender, and handedness matched HC participants, who were screened for mental disorders with the German version of the Structural Clinical Interview for DSM-IV-R Non-Patient Edition (SCID), participated in the study. Due to the insufficient number (i.e., <30) of illusion or no-illusion trials following the EEG artifact rejection, data of nine SCZ (mean illusion rate = 71.43%) and seven HC (mean illusion rate = 81.53%) were excluded from further analyses. Participants were excluded due to the following reasons: lack of illusion perception (i.e., <30 illusion trials; *N* = 2 SCZ, *N* = 1 HC), lack of no-illusion perception (i.e., <30 no-illusion trials; *N* = 7 SCZ, *N* = 6 HC). The illusion rates for the excluded SCZ and HC did not significantly differ (Mann–Whitney *U* test = 20, *p* = 0.223). For the final data analysis, the 15 best matching HC were selected (based on age, education, gender, handedness). All patients met DSM-IV-TR criteria for SCZ. The psychiatric diagnosis was assessed by a senior psychiatrist at the recruiting institution. The study was conducted in accordance with the Declaration of Helsinki and approved by the ethics committee of the Charité – Universitätsmedizin Berlin. All participants provided written informed consent, had normal hearing, normal or corrected to normal vision, and no neurological disorders, alcohol or substance abuse. A random sample of 40% of all participants underwent a multi-drug screening and all of those tested had negative results. Severity of symptoms in SCZ was assessed with the Positive and Negative Syndrome Scale (PANSS; Kay et al., 1987). To test cognitive performance, the Brief Assessment of Cognition in Schizophrenia (BACS) was assessed (Keefe et al., 2004). **Table 1** provides an overview on demographic data, cognitive performance, and clinical scores of the study participants.

**Table 1:** Demographic data, positive and negative syndromes, and cognitive scores in the study participants.

	Patients		Controls		Statistics	
	Mean	*SD*	Mean	*SD*	*t*-values	*p*-values
Age (years)	33.87	7.23	36.13	7.91	-0.819	0.420
Education (years)	10.93	1.44	10.87	1.81	0.112	0.912
Illness duration (years)	9	4.8	–	–	–	–
Chlorpromazine equivalent level (daily dosage/mg)	398.73	167.68	–	–	–	–
	***N***		***N***			
Gender (m/f)	12/3		12/3		–	–
Handedness (r/l)	13/2		13/2		–	–
Antipsychotic Medications	15		–		–	–
Co-medication^∗^	4		–		–	–
BACS						
Verbal memory	42.93	11.02	44.20	9.70	–0.334	0.741
Digit	18.87	3.25	20.27	3.90	-1.068	0.295
Motor	65.80	14.06	72.53	9.93	-1.515	0.141
Fluency	49.13	12.08	50.47	15.05	-0.268	0.791
Symbol coding	54.67	11.84	54.73	14.80	-0.014	0.989
ToL	16.87	3.14	17.40	2.26	-0.534	0.597
Total score	248.27	41.65	259.60	36.89	-0.789	0.437
PANSS						
Negative	18.47	3.16	–	–	–	–
Positive	17.40	2.53	–	–	–	–
General	38.20	3.12	–	–	–	–
Total score	74.07	8.81	–	–	–	–

The table depicts demographic data and BACS scores for SCZ and HC, as well as PANSS scores for SCZ. BACS, Brief Assessment of Cognition in Schizophrenia; PANSS, Positive and Negative Syndrome Scale. *Co-medication of antipsychotics and mood stabilizers.

### Experimental Design

**FIGURE 1:**
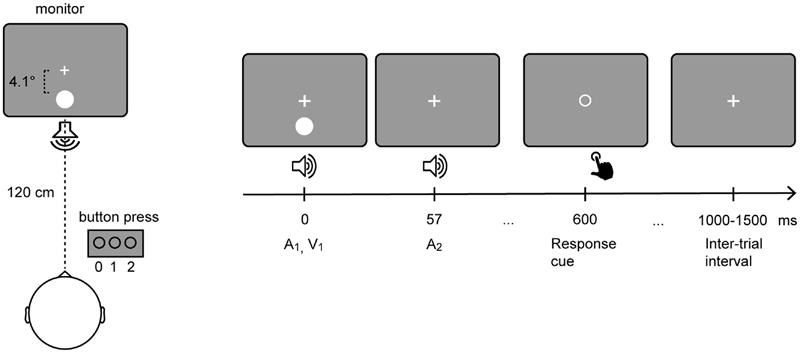
**Setup of the sound-induced flash illusion paradigm.** Participants fixated a central white cross while being presented with stimuli of the SIFI paradigm (**Left**). In a critical SIFI trial (i.e., A_2_V_1_) a single flash presented alongside two rapidly repeating tones is either perceived as one or two flashes. (**Right**) depicts the timeline of the critical SIFI trial. The visual stimulus and the first auditory stimulus are presented simultaneously. The second auditory stimulus is presented 57 ms after the onset of the first stimulus. Six hundred milliseconds after the onset of the first stimulus, the fixation cross is replaced by a response cue, which comprised an empty circle that is presented in the center of the screen.

The experiment was conducted in a sound-attenuated electrically shielded chamber. Stimuli were presented on a CRT monitor with a background luminance of 21 cd/m^2^. Six stimulus combinations were presented: A_0_V_1_, A_0_V_2_, A_1_V_1_, A_2_V_0_, A_2_V_1_, A_2_V_2_, where the indexed numbers denote the number of auditory (A) and visual (V) inputs. Participants fixated a central white cross while being presented with stimuli of the SIFI paradigm (**Figure 1**). The participants’ task was to report the number of perceived visual stimuli by pressing a button with the index, middle, or ring finger of their right hand to indicate whether they perceived 0, 1, or 2 flashes, respectively. Each visual stimulus was presented for 10 ms and consisted of a white disk subtending 1.6° with a luminance of 89 cd/m^2^. Visual stimuli were presented at 4.1° centrally below the fixation cross. Each auditory stimulus was presented for 7 ms and consisted of a 73 dB (SPL) 1000 Hz sine wave tone. Auditory stimuli were presented from a central speaker below the screen. Three hundred SIFI trials and 150 trials per control condition were presented in random order in eight blocks.

### Analysis of Behavioral Data

For all stimulus combinations, the numbers of reported zero, one or two flashes were calculated relative to the total number of trials in each condition (Supplementary Figure 1). For the critical A_2_V_1_ condition, illusion rates were calculated as the percentage of two perceived flashes in relation to the total number of A_2_V_1_ trials. Within SCZ these values were related to the psychopathology scores (PANSS) by using Pearson correlations. To statistically control for the influence of anti-psychotic medication, medication dosage was converted to chlorpromazine equivalent level (Gardner et al., 2010) and entered as covariate to partial correlation analyses in the patient group. We calculated *t*-tests for each condition to compare the behavioral results between SCZ and HC. To account for multiple comparisons, the statistical outcome was Bonferroni corrected.

### Acquisition and Preprocessing of EEG Data

EEG was recorded using a 128-electrodes active system (EasyCap, Herrsching, Germany), including one horizontal and one vertical EOG electrode to monitor eye movements. Data were recorded against nose reference with a pass band (0.016–250 Hz) and digitized at a sampling rate of 1000 Hz. Preprocessing and oﬄine data analysis were performed using EEGlab (Delorme and Makeig, 2004), Fieldtrip (Oostenveld et al., 2011), and custom-made Matlab scripts (MathWorks, Natick, MA, USA). In our analyses we followed previously reported procedures (Gross et al., 2013; Gross, 2014; Keil J. et al., 2014). Data were oﬄine high-pass filtered (1 Hz, FIR), low-pass filtered (125 Hz, FIR), and notch-filtered (49.0–51 Hz, 4th order two-pass Butterworth filter). Moreover, data were down-sampled to 500 Hz.

For the data analysis, epochs of 4 s (-1 to 3 s around the onset of the first auditory stimulus) were extracted and those containing muscular artifacts were rejected by visual inspection. In total, 1050 trials were presented. Trials containing remaining artifacts with amplitudes of ±100 μV were rejected automatically. After artifact rejection, we used on average 960.13 (*SD* = 53.40) trials per individual in the SCZ group for further analyses. In the HC group we used on average 952.73 (*SD* = 62.24) trials for further analyses. For the multisensory A_2_V_1_ trials, we used on average 152.93 (*SD* = 67.61) illusion trials and 104 (*SD* = 54.73) no-illusion trials per individual in the SCZ group. In the HC group we used on average 145.47 (*SD* = 56.25) illusion trials and 113.53 (*SD* = 56.08) no-illusion trials. For the statistical analysis, the number of A_2_V_1_ trials was equalized between conditions, i.e., illusion and no-illusion, using the lowest number of available trials in either condition. This was done separately for each individual.

Independent component analyses were conducted to correct for EOG and ECG artifacts (extended runica; Lee et al., 1999). On average, 14.73 ± 5.06 (*SD*) independent components for SCZ and 15.67 ± 6.17 independent components for HC were rejected. Next, noisy electrodes were interpolated using spherical interpolation (average SCZ = 1.40 ± 1.50 electrodes; HC = 1.27 ± 1.58 electrodes). Finally, the epoched data were re-referenced to common average and the epoch mean was removed from each epoch.

For the ERP analysis of multisensory A_2_V_1_ trials, data of SCZ and HC were filtered (35 Hz low-pass, 4th order two-pass Butterworth filter), averaged over trials and a baseline correction was performed (-500 to 0 ms prior to stimulus onset). ERPs were analyzed in a time interval from 0 to 400 ms following stimulus onset. Since no prior EEG study has investigated the SIFI in SCZ, we did not have ad hoc hypotheses about the ERP components that could be altered in this patient group. For this reason, we performed non-parametric tests with cluster-based correction for multiple comparisons (Maris and Oostenveld, 2007; Maris, 2012). This was done separately between the different percepts (factor Perception: illusion vs. no-illusion trials), between groups (factor Group: SCZ vs. HC), and for the Perception by Group interaction (i.e., by comparing the differences between illusion vs. no-illusion trials between SCZ and HC). Significant interactions were followed up by *post hoc t*-tests. If an effect was significant, Cohen’s *d* was calculated, as marker of the effect size (**Table 2**).

**Table 2:** Significant findings of the non-parametric tests.

Multisensory A_2_V_1_ trials: non-parametric tests with the factors Perception (illusion vs. no-illusion) and Group (SCZ vs. HC)				
	**Time**	***t*-values**	**Cluster**	**Cohen’s *d***
	**interval**	**(RMS)**	***p*-values**	
	
Perception	0.11–0.16 s	1.766	–	0.456
Group	0.09–0.19 s	2.002	0.005	0.731
Interaction	0.11–0.16 s	2.253	0.047	0.823

**Visual trials: non-parametric tests with the factors Condition (A_0_V_1_ vs. A_0_V_2_) and Group (SCZ vs. HC)**				

	**Time**	***t*-values**	**Cluster**	**Cohen’s *d***
	**interval**	**(RMS)**	***p*-values**	
	
Condition	0.16–0.27 s	1.924	0.006	0.497
Group	0.16–0.27 s	1.141	–	0.417
Interaction	0.16–0.27 s	1.462	–	0.534

**Auditory A_2_V_0_ trials: non-parametric tests with the factor Group (SCZ vs. HC)**				

Group	n.s.			

*The table summarizes the results of the non-parametric tests with cluster-based correction for multiple comparisons, which have been conducted for multisensory (upper), visual (middle) and auditory (lower) trials. For each time interval, *t*-values (RMS), significant cluster *p*-values and Cohen’s *d* are reported.*

To test if there are differences between the processing of multisensory and unisensory stimuli, unisensory visual A_0_V_1_ and A_0_V_2_ trials, as well as unisensory auditory A_2_V_0_ trials were analyzed accordingly. Again, we performed non-parametric tests with cluster-based correction for multiple comparisons between conditions (factor Condition: A_0_V_1_ vs. A_0_V_2_), between groups (factor Group: SCZ vs. HC), and for the Condition by Group interaction (i.e., by comparing the differences between A_0_V_1_ vs. A_0_V_2_ trials between SCZ and HC). For the auditory A_2_V_0_ trials, a similar comparison between groups was conducted. Previous studies on the SIFI have used the additive approach to investigate multisensory interactions in ERPs (Mishra et al., 2008, 2010). In this approach, ERPs to multisensory stimuli are linearly combined with activity from trials in which no stimuli were presented (so called ”No-Stim” trials, Busse and Woldorff, 2003; Senkowski et al., 2007). These combined responses, i.e., multisensory AV plus ‘No-Stim’ ERPs, are then compared with the combined ERPs to the respective unisensory stimuli, e.g., unisensory A plus unisensory V. In the present study, we did not include ‘No-Stim’ events and did therefore not apply the additive approach.

### Time-Frequency Analysis

In agreement with the finding of a previous study in healthy individuals (Mishra et al., 2007), the analysis of neural oscillations explicitly focused on the examination of beta/gamma band power over the occipital cortex. To this end, time-frequency representations (TFRs) of single trials were calculated for each subject and for each relevant condition (i.e., multisensory: A_2_V_1_, unisensory: A_0_V_1_, A_0_V_2_, A_2_V_0_). Morlet Wavelets with a width of seven cycles per frequency were used to calculate spectral estimates at each point of the time-frequency window ranging from -1000 to 1500 ms (10 ms steps) in the time domain and 2 to 100 Hz (1 Hz steps) in the frequency domain. TFRs were baseline-corrected with respect to the interval ranging from -500 to -100 ms prior to stimulus onset (absolute baseline). In accordance with Mishra et al. (2007), for the statistical analysis individual mean 25 to 35 Hz total power in the 100 to 150 ms interval and individual mean 32 to 40 Hz total power in the 200 to 240 ms interval was extracted from a sensor-level ROI consisting of 24 electrodes. The ROI closely matched the 25–35 Hz power topography reported by Mishra et al. (2007). Individual mean power was then submitted to repeated measures 2 × 2 ANOVAs with the within-subject factor Perception (illusion vs. no-illusion) and the between-subjects factor Group (SCZ vs. HC). To test if there are differences between the processing of multisensory and unisensory stimuli, an analogous analysis was carried out for unisensory visual stimuli with the within-subject factor Condition (A_0_V_1_ vs. A_0_V_2_) and the between-subjects factor Group (SCZ vs. HC). Significant main effects or interactions were followed up by *post hoc t*-tests. Finally, for the unisensory auditory condition (i.e., A_2_V_0_) a *t*-test was used to test the main effect of Group.

## Results

### Behavioral Data

Schizophrenia reported perceiving two flashes in 55.7% of the critical A_2_V_1_ trials, HC in 55.4% of the A_2_V_1_ trials. The illusion rates did not significantly differ between SCZ and HC [*t*(28) = 0.028, *p* = 0.978]. Moreover, the behavioral analyses for the other stimulus types revealed that participants correctly reported the number of presented visual and auditory inputs (Supplementary Figure 1). Furthermore, the comparison of the percepts in the control conditions did not reveal any group differences (Supplementary Figure 1). The partial correlation analyses between SIFI illusion rates and clinical symptoms (i.e., PANSS subscale scores), in which medication dose in SCZ served as control variable, were not significant (all *p*-values > 0.2).

### Event-Related Potentials

**FIGURE 2:**
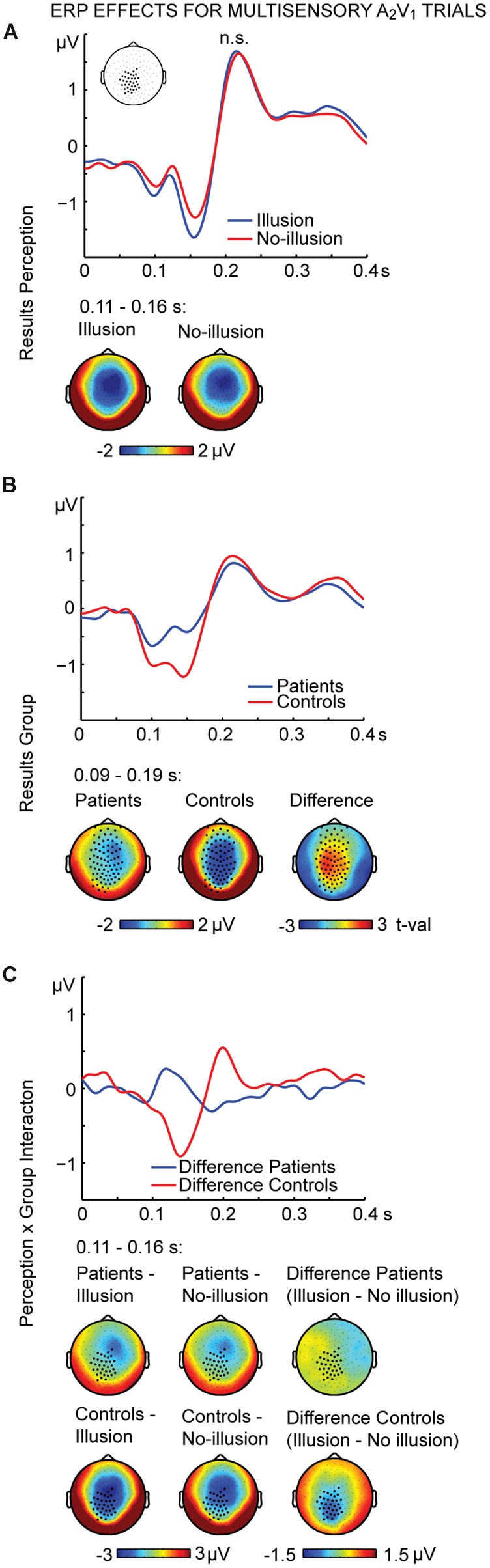
**ERP effects of multisensory A_2_V_1_ trials.** Outcome of the non-parametric tests with cluster-based correction for multiple comparisons between Perceptions (illusion vs. no-illusion), between Groups (SCZ vs. HC), and for the Perception differences between Groups (i.e., the Perception by Group interaction). The upper planes in panels **(A–C)** illustrate the ERP results for Perception **(A)**, the main effect of Group **(B)**, and the Perception by Group interaction **(C)**. The lower planes depict topographic maps for the observed results with highlighted significant cluster electrodes. Time-point 0 indicates the onset of the first auditory and visual stimulus.

The non-parametric dependent samples test for multisensory A_2_V_1_ trials, which was conducted for all electrodes, revealed no significant differences between illusion and no-illusion A_2_V_1_ trials (**Figure 2A**). This observation was somewhat surprising and motivated us to conduct an exploratory running non-parametric dependent samples test to examine whether there are ERP differences between illusion and no-illusion A_2_V_1_ trials in HC only, as previously reported (Mishra et al., 2007). We found significant differences between illusion and no-illusion trials in the 102 to 172 ms and 314 to 384 ms intervals, which indicate perception-related modulations of the visual N1 and P3 components specifically in healthy individuals (Supplementary Figure 2). The non-parametric independent samples test between SCZ and HC revealed significant group differences between 88 and 186 ms. More negative amplitudes in HC compared to SCZ were found at a central cluster comprising of 69 electrodes (**Figure 2B**; **Table 2**, upper panel). Notably, the non-parametric independent samples test between the Perception differences for SCZ and HC revealed a significant Perception by Group interaction (**Figure 2C**). The amplitude differences between illusion and no-illusion trials were significantly larger for HC than for SCZ, especially between 106 and 164 ms. The interaction patterns were most robust at a cluster comprising of 34 central to posterior electrodes. Follow-up *post hoc t*-tests revealed no differences between illusion compared to no-illusion trials in SCZ [*t*(14) = 0.980, *p* = 0.344]. However, there were significant differences between illusion compared to no-illusion trials in HC [*t*(14) = -5.220, *p* = 0.000]. Further testing revealed group differences in the 106 to 164 ms interval for illusion trials [*t*(28) = 3.796, *p* = 0.001], as well as for no-illusion trials [*t*(28) = 2.131, *p* = 0.042]. The ERP amplitudes for both percepts were lower in SCZ compared to HC.

**FIGURE 3:**
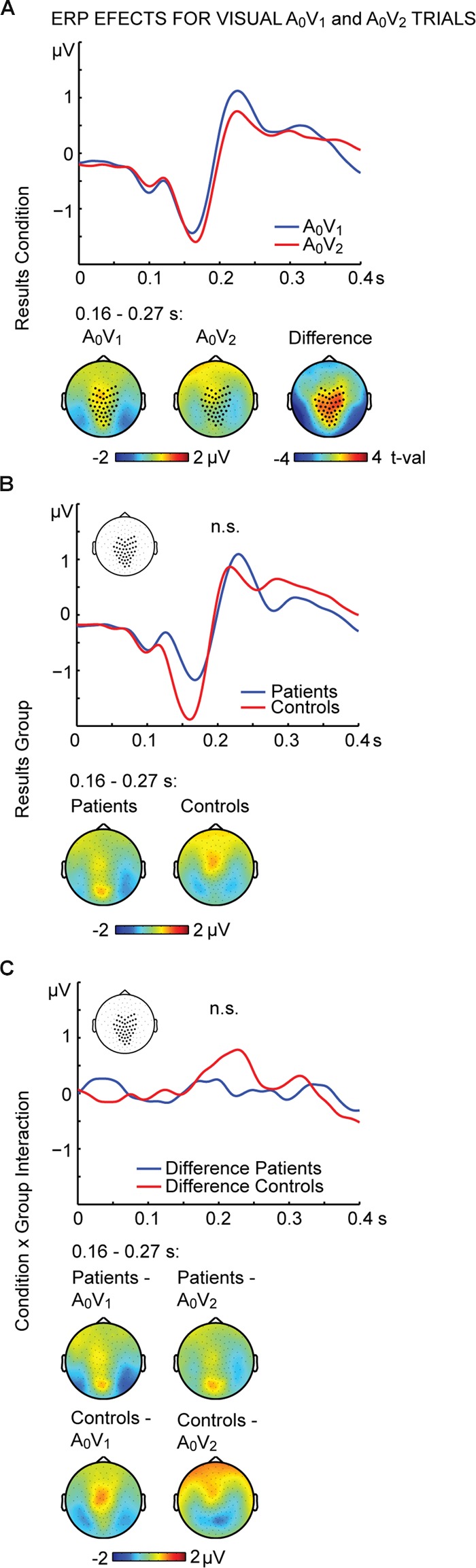
**ERP effects of visual A_0_V_1_ and A_0_V_2_ trials.** Outcome of the non-parametric tests with cluster-based correction for multiple comparisons between Conditions (A_0_V_1_ vs. A_0_V_2_) between Groups (SCZ vs. HC), and for the Condition differences between Groups (i.e., the Condition by Group interaction). The upper planes in panels **(A–C)** illustrate significant main effects of Condition **(A)**, the ERP results for Group **(B)** and the Condition by Group interaction **(C)**. The lower planes depict topographic maps for the observed effects with highlighted significant cluster electrodes. Time-point 0 indicates the onset of the first visual stimulus.

To further examine the effect of visual stimulation alone we conducted non-parametric tests for the unisensory visual conditions (i.e., A_0_V_1_ vs. A_0_V_2_). For the interval between 164 and 268 ms we found significant differences between conditions (**Figure 3A**; **Table 2**, middle panel). The amplitudes in the A_0_V_2_ trials compared to A_0_V_1_ trials were more negative at a cluster comprising of 46 central and posterior electrodes. No main effect of Group or Perception by Group interaction was found (**Figures 3B,C**). Finally, we compared ERPs to unisensory auditory trials (i.e., A_2_V_0_) between groups. This analysis revealed no significant group differences (Supplementary Figure 3).

### Time-Frequency Analysis

**FIGURE 4:**
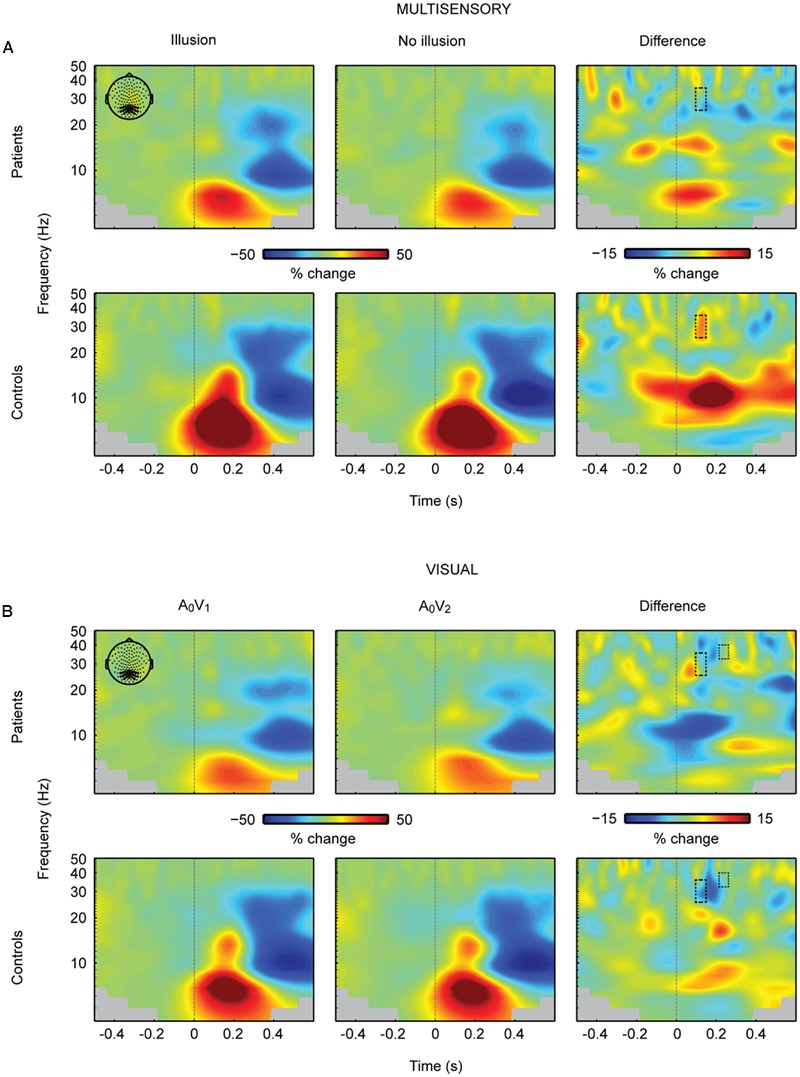
**Total oscillatory power over occipital cortex. (A)** Time-frequency representations (TFRs) at occipital electrodes in response to the critical multisensory A_2_V_1_ trials. In the early time-frequency window (100–150 ms, 25–35 Hz; highlighted in a box) oscillatory response patterns accompanying illusion and no-illusion trials were different between groups. In the control group, the 25–35 Hz total power was stronger in illusion compared to no-illusion trials. No such difference was found in SCZ. No effects were found in the late time-frequency window (200–240 ms, 25–35 Hz). **(B)** TFRs at occipital electrodes in response to unisensory visual trials. Total power in the early and late time-frequency window (each highlighted in a box) was significantly higher in the A_0_V_2_ condition compared to the A_0_V_1_ condition. No Group differences (i.e., SCZ vs. HC) or Group by Condition interactions were found. Time-point 0 indicates the onset of the first auditory and first visual stimulus.

The analysis of neural oscillations focused on occipital 25–35 Hz power between 100 and 150 ms and occipital 32–40 Hz power between 200 and 240 ms. These time-frequency windows were selected based on findings of multisensory interactions in the SIFI obtained by Mishra et al. (2007). The repeated measures 2 × 2 ANOVA for multisensory A_2_V_1_ trials in the 100 to 150 ms time-frequency window revealed no main effect of Perception [*F*(1,28) = 0.813, *p* = 0.375] or Group [*F*(1,28) = 1.133, *p* = 0.296]. However, a significant Perception by Group interaction for the early time-frequency window between 100 and 150 ms was found [*F*(1,28) = 4.940, *p* = 0.035; **Figure 4A**]. Follow-up *t*-tests revealed significantly larger 25–35 Hz power in illusion compared to no-illusion trials in HC [*t*(14) = 2.502, *p* = 0.025], but no power differences between illusion and no-illusion trials in SCZ [*t*(14) = -0.845, *p* = 0.412]. The analysis of the 32-40 Hz power in the 200 to 240 ms time-frequency window did not reveal any significant effects. Analogous to multisensory trials, we calculated power analyses for unisensory visual trials (i.e., A_0_V_1_ vs. A_0_V_2_; **Figure 4B**). The analyses of the 100–150 ms time-frequency window revealed a significant main effect of Condition [*F*(1,28) = 6.903, *p* = 0.014], due to higher power in A_0_V_2_ compared to A_0_V_1_ trials. No main effect of Group [*F*(1,28) = 1.834, *p* = 0.187] or Condition by Group interaction [*F*(1,28) = 1.418, *p* = 0.244] was found. The analyses of the 200–240 ms time-frequency window also revealed a significant main effect of Condition [*F*(1,28) = 4.521, *p* = 0.042], due to higher power in A_0_V_2_ compared to A_0_V_1_ trials. No main effect of Group [*F*(1,28) = 2.926, *p* = 0.098], or Condition by Group interaction [*F*(1,28) = 0.034, *p* = 0.856] was found. Finally, we compared 25–35 Hz power in unisensory auditory stimuli (i.e., A_2_V_0_; Supplementary Figure 4) between groups. This analysis did not reveal any significant differences between SCZ and HC [*t*(28) = 1.240, *p* = 0.225 for the 100–150 ms time-frequency window; *t*(28) = 1.900, *p* = 0.068 for the 200–240 ms time-frequency window]. For exploratory purposes we also analyzed alpha-band power between 100 and 300 ms in the frequency range of 8–13 Hz. The 2 × 2 ANOVA did not reveal any significant effects.

## Discussion

In this study, we examined multisensory integration in SCZ and HC using the SIFI paradigm. Our study revealed several interesting findings. Contrary to our hypothesis, on the behavioral level, SCZ and HC did not significantly differ in the perception rates of the SIFI. However, SCZ compared to HC showed reduced amplitudes and diminished ERP differences between illusion and no-illusion trials. Moreover, SCZ lacked an early enhancement of 25–35 Hz total power for illusion compared to no-illusion SIFI trials, which was observed in HC.

### Sound-Induced Flash Illusion Rates Are Comparable between Patients and Healthy Control Participants

On average, both SCZ and HC perceived the SIFI in about 55% of the critical A_2_V_1_ trials. Additionally, there were no perceptual differences between SCZ and HC in the control conditions. In these conditions, both SCZ and HC reported the correct numbers of visual stimuli in 73–95% of trials, indicating that both groups maintained attention throughout the experiment. Hence, the behavioral data suggest no substantial perceptual alterations in SCZ. Given that previous studies have reported aberrant multisensory processing in SCZ (de Boer-Schellekens et al., 2014), the lack of perceptual alterations in the current study is somewhat surprising. In unisensory processing, aberrant neural processing often coincides with altered perception, which is reflected in behavioral deficits (Senkowski and Gallinat, 2015; Uhlhaas and Singer, 2015). Therefore, we expected that differences in multisensory processing in the SIFI would affect the perceptual outcome between the groups. However, it is possible that SCZ show different multisensory processing patterns compared to HC, which still lead to the same perceptual outcome. These different multisensory processing patters might be indicative of compensatory mechanisms, altered signal fidelity or unspecific stimulus processing deficits.

Nevertheless, other studies did also not find differences in the perception of multisensory stimuli between SCZ and HC (Surguladze et al., 2001; Pearl et al., 2009; Martin et al., 2013; Wynn et al., 2014; Roa Romero et al., 2016a). For example, examining the McGurk illusion, Martin et al. (2013), as well as Roa Romero et al. (2016a) observed comparable illusion rates in SCZ and HC. Together with the present findings, these studies suggest that aberrant multisensory processing is not necessarily linked to aberrant multisensory perception. In summary, our behavioral data did not reveal alterations in SIFI perception rates in SCZ.

### Patients with Schizophrenia Show Diminished ERP Effects in the Sound-Induced Flash Illusion Paradigm

Within HC, we observed larger ERP amplitudes around 140 ms and around 350 ms to illusion compared to no-illusion multisensory trials. This observation is comparable with previous findings in healthy individuals (Mishra et al., 2007; Keil A. et al., 2014). In the current study, HC compared to SCZ showed larger ERP amplitudes, irrespective of whether the critical SIFI trials were perceived as illusion or no-illusion. This indicates general group differences in the processing of multisensory stimuli. The ERP amplitudes around 140 ms were larger in HC compared to SCZ. Reduced EEG data quality in patients could have contributed to the reduced amplitudes. However, reduced ERP amplitudes in SCZ have been also found in numerous previous studies (e.g., Stone et al., 2011; Onitsuka et al., 2013; Stekelenburg et al., 2013). Furthermore, the number of rejected EEG trials in the analysis did not differ between groups. After the artifact rejection, there were on average 960 trials in the SCZ group and 953 trials in the control group. Therefore, we propose that the reduced ERP amplitudes in SCZ reflect processing alterations in patients and are not due to group differences in the EEG data quality. Taken together, reduced amplitudes in SCZ compared to HC following multisensory stimulation are found in early ERP components.

Importantly, we observed differences in multisensory integration effects, i.e., illusion vs. no-illusion trials, between groups. The integration effects, which were found around 135 ms after the onset of the first auditory and visual stimulus, were more robust in HC compared to SCZ (**Figure 2**). In unisensory processing, aberrant neural processing in SCZ often coincides with altered perception (Senkowski and Gallinat, 2015; Uhlhaas and Singer, 2015). Therefore, we expected that differences in multisensory processing in the SIFI would also affect the perceptual outcome. However, this was not the case. Recently, Stekelenburg et al. (2013) and Roa Romero et al. (2016b) also reported group differences in multisensory ERPs without showing effects in behavioral data. In an interesting framework on multisensory processing, Bizley et al. (2016) suggested different stages of multisensory integration, in which the initial integrative processing can occur, at least partially, independent of later perception-related processing. In line with this proposal, the initial processing and integration could be disturbed in the SIFI trials in SCZ, whereas the later perceptual stages might be still intact. Thus, there could be later mechanisms in the perception of multisensory stimuli in SCZ that compensate for earlier alterations in sensory processing by recruiting additional brain networks or additional regions within the same network (Rentrop et al., 2011). However, the assumption that compensatory mechanisms might support multisensory perception in SCZ requires further empirical testing. For instance, transcranial magnetic stimulation (TMS) could be applied at early and late time intervals following the presentation of SIFI trials. If there were compensatory mechanisms in SCZ then TMS applied specifically at late intervals should affect perception in patients. In sum, our study revealed altered processing of multisensory stimuli in SCZ. However, this did not affect the SIFI perception rate.

To better understand the observation of reduced multisensory processing in SCZ, the ERP findings in the critical multisensory SIFI trials can be compared to the findings in unisensory trials. Across groups, the ERP amplitudes of unisensory visual stimuli around 215 ms were larger in trials with two visual inputs compared to trials with one visual input. This effect was found in both groups, which might indicate that unisensory processing differences between 1 and 2 visual stimuli are comparable between SCZ and HC and that aberrant processing predominantly occurs in the processing of multisensory stimuli. Previous studies also found evidence for alterations in multisensory processing, but not in unisensory processing in SCZ (Ross et al., 2007). For example, Ross et al. (2007) found specific deficits in the ability of SCZ to integrate visual and auditory speech. SCZ experienced less benefit from visual articulation when they tried to comprehend speech under noisy environmental conditions, whereas their performance in unisensory auditory speech perception remained fully intact. This could imply that sensory processing in SCZ is particularly impaired when stimuli are presented in a multisensory way. The integration process of multisensory information might be more complex than the processing of information that is presented to only one sense.

### Schizophrenia Patients Lack an Enhancement of 25–35 Hz Power to Audiovisual Illusion Trials

In the critical multisensory trials, HC displayed higher 25–35 Hz total power to illusion compared to no-illusion trials in the time window of 100 to 150 ms after stimulus onset. The observation of enhanced occipital power in healthy individuals replicates the previous finding by Mishra et al. (2007). Interestingly, the current study revealed that SCZ displayed no power differences at 25–35 Hz between illusion compared to no-illusion trials. Specifically, our study showed that patients lack an enhancement of neural oscillations over the occipital cortex to illusion trials. Interestingly, our data did not show general group differences for the 25–35 Hz responses to multisensory and unisensory visual or unisensory auditory stimuli. It might be that the absence of significant main effects between groups relates to a lack of statistical power. Indeed, previous studies have reported diminished evoked beta band (13–30 Hz) and gamma band (>30 Hz) oscillations in SCZ (Gallinat et al., 2004; Leicht et al., 2010, 2015; Lenz et al., 2011; Keil et al., 2016). For example, Leicht et al. (2010) investigated auditory evoked gamma band oscillations in SCZ and found a significant reduction of power in SCZ compared to HC around 50 ms after stimulus presentation. Keil et al. (2016) recently replicated this effect. Another study by Lenz et al. (2011) examined evoked gamma band responses in SCZ during an auditory oddball-paradigm and reported diminished responses to standard and deviant stimuli in SCZ around 40 to 90 ms after stimulus onset. These studies support our finding of diminished 25–35 Hz oscillations to illusory multisensory SIFI trials in SCZ. Our findings might indicate an early binding deficit in SCZ (Singer and Gray, 1995; Engel et al., 2001). Taken together with previous findings, our study suggests that altered 25–35 Hz oscillatory responses reflect aberrant multisensory processing in SCZ.

## Conclusion

In this study, we compared multisensory processing of the SIFI paradigm between HC and SCZ. We found similar multisensory illusion rates between groups. Importantly, our analysis of ERP data revealed altered multisensory integration effects in SCZ compared to HC. Our findings are in agreement with recent ERP studies showing aberrant multisensory processing in SCZ. Moreover, our study complements recent reports of altered multisensory integration in the McGurk illusion, suggesting processing differences between SCZ and HC in various prominent multisensory illusion paradigms. Surprisingly, while differences in neural response patterns are relatively consistently observed, a number of studies revealed no alterations in behavior. Hence, further studies are required to uncover the precise associations between altered multisensory processing and behavioral as well as perceptual outcome in SCZ. Our analysis of neural oscillations revealed reduced occipital 25–35 Hz responses to illusory multisensory SIFI trials in SCZ. Thus far, neural oscillations during multisensory processing have been rarely investigated in SCZ. Previously, various studies have reported altered neural oscillations during unisensory processing in this patient group. Therefore, our observation of altered neural oscillations during multisensory processing provides further support for the notion that dysfunctional neural oscillations signify aberrant integrative sensory processing in SCZ.

## Author Contributions

JB conceived the experiment, recorded data, analyzed data, and wrote the manuscript. YR conceived the experiment, recorded data, and wrote the manuscript. JK conceived the experiment, analyzed data, and wrote the manuscript. MK analyzed data and wrote the manuscript. MN discussed the data and wrote the manuscript. JG conceived the experiment and wrote the manuscript. DS conceived the experiment, discussed data, and wrote the manuscript.

## Conflict of Interest Statement

The authors declare that the research was conducted in the absence of any commercial or financial relationships that could be construed as a potential conflict of interest.

## References

[B1] Andreasen N. C. (2000). Schizophrenia: the fundamental questions. *Brain Res. Brain Res. Rev.* 31 106–112. 10.1016/S0165-0173(99)00027-2 10719138

[B2] Balz J. Keil J. Roa Romero Y. Mekle R. Schubert F. Aydin S. (2016). GABA concentration in superior temporal sulcus predicts gamma power and perception in the sound-induced flash illusion. *Neuroimage* 125 724–730. 10.1016/j.neuroimage.2015.10.087 26546865

[B3] Bhattacharya J. Shams L. Shimojo S. (2002). Sound-induced illusory flash perception: role of gamma band responses. *Neuroreport* 13 1727–1730. 10.1097/00001756-200210070-00007 12395112

[B4] Bizley J. K. Maddox R. K. Lee A. K. C. (2016). Defining auditory-visual objects: behavioral tests and physiological mechanisms. *Trends Neurosci.* 39 74–85. 10.1016/j.tins.2015.12.007 26775728 PMC4738154

[B5] Busse L. Woldorff M. G. (2003). The ERP omitted stimulus response to “no-stim” events and its implications for fast-rate event-related fMRI designs. *Neuroimage* 18 856–864. 10.1016/S1053-8119(03)00012-0 12725762

[B6] Capa R. L. Duval C. Z. Blaison D. Giersch A. (2014). Patients with schizophrenia selectively impaired in temporal order judgments. *Schizophr. Res.* 156 51–55. 10.1016/j.schres.2014.04.001 24768441

[B7] Chen Y. Norton D. Stromeyer C. III (2014). Prolonged temporal interaction for peripheral visual processing in schizophrenia: evidence from a three-flash illusion. *Schizophr. Res.* 156 190–196. 10.1016/j.schres.2014.04.018 24814873 PMC4096562

[B8] de Boer-Schellekens L. Stekelenburg J. J. Maes J. P. van Gool A. R. Vroomen J. (2014). Sound improves diminished visual temporal sensitivity in schizophrenia. *Acta Psychol.* 147 136–142. 10.1016/j.actpsy.2013.06.013 23896561

[B9] Delorme A. Makeig S. (2004). EEGLAB: an open source toolbox for analysis of single-trial EEG dynamics including independent component analysis. *J. Neurosci. Methods* 134 9–21. 10.1016/j.jneumeth.2003.10.009 15102499

[B10] Engel A. K. Fries P. Singer W. (2001). Dynamic predictions: oscillations and synchrony in top-down processing. *Nat. Rev. Neurosci.* 2 704–716. 10.1038/35094565 11584308

[B11] Foucher J. R. Lacambre M. Pham B. T. Giersch A. Elliott M. A. (2007). Low time resolution in schizophrenia Lengthened windows of simultaneity for visual, auditory and bimodal stimuli. *Schizophr. Res.* 97 118–127. 10.1016/j.schres.2007.08.013 17884350

[B12] Freedman R. Waldo M. Bickford-Wimer P. Nagamoto H. (1991). Elementary neuronal dysfunctions in schizophrenia. *Schizophr. Res.* 4 233–243. 10.1016/0920-9964(91)90035-P 1645590

[B13] Gallinat J. Winterer G. Herrmann C. S. Senkowski D. (2004). Reduced oscillatory gamma-band responses in unmedicated schizophrenic patients indicate impaired frontal network processing. *Clin. Neurophysiol.* 115 1863–1874. 10.1016/j.clinph.2004.03.013 15261865

[B14] Gardner D. M. Murphy A. L. O’Donnell H. O. Centorrino F. Baldessarini R. J. (2010). International consensus study of antipsychotic dosing. *Am. J. Psychiatry* 167 686–693. 10.1176/appi.ajp.2009.09060802 20360319

[B15] Giersch A. Lalanne L. van Assche M. Elliott M. A. (2013). On disturbed time continuity in schizophrenia: an elementary impairment in visual perception? *Front. Psychol.* 4:281 10.3389/fpsyg.2013.00281 PMC3664782 23755027

[B16] Gross J. (2014). Analytical methods and experimental approaches for electrophysiological studies of brain oscillations. *J. Neurosci. Methods* 228 57–66. 10.1016/j.jneumeth.2014.03.007 24675051 PMC4007035

[B17] Gross J. Baillet S. Barnes G. R. Henson R. N. Hillebrand A. Jensen O. (2013). Good practice for conducting and reporting MEG research. *Neuroimage* 65 349–363. 10.1016/j.neuroimage.2012.10.001 23046981 PMC3925794

[B18] Javitt D. C. Freedman R. (2015). Sensory processing dysfunction in the personal experience and neuronal machinery of schizophrenia. *Am. J. Psychiatry* 172 17–31. 10.1176/appi.ajp.2014.13121691 25553496 PMC4501403

[B19] Kay S. R. Fiszbein A. Opler L. A. (1987). The positive and negative syndrome scale (PANSS) for schizophrenia. *Schizophr. Bull.* 13 261–276. 10.1093/schbul/13.2.261 3616518

[B20] Keefe R. S. E. Goldberg T. E. Harvey P. D. Gold J. M. Poe M. P. Coughenour L. (2004). The brief assessment of cognition in schizophrenia: reliability, sensitivity, and comparison with a standard neurocognitive battery. *Schizophr. Res.* 68 283–297. 10.1016/j.schres.2003.09.011 15099610

[B21] Keil A. Debener S. Gratton G. Junghöfer M. Kappenman E. S. Luck S. J. (2014). Committee report: publication guidelines and recommendations for studies using electroencephalography and magnetoencephalography. *Psychophysiology* 51 1–21. 10.1111/psyp.12147 24147581

[B22] Keil J. Müller N. Hartmann T. Weisz N. (2014). Prestimulus beta power and phase synchrony influence the sound-induced flash illusion. *Cereb. cortex* 24 1278–1288. 10.1093/cercor/bhs409 23300109

[B23] Keil J. Roa Romero Y. Balz J. Henjes M. Senkowski D. (2016). Positive and negative symptoms in schizophrenia relate to distinct oscillatory signatures of sensory gating. *Front. Hum. Neurosci.* 10:104 10.3389/fnhum.2016.00104 PMC4789458 27014035

[B24] Lee K.-H. Williams L. M. Breakspear M. Gordon E. (2003). Synchronous gamma activity: a review and contribution to an integrative neuroscience model of schizophrenia. *Brain Res. Brain Res. Rev.* 41 57–78. 10.1016/S0165-0173(02)00220-5 12505648

[B25] Lee T.-W. Girolami M. Sejnowski T. J. (1999). Independent component analysis using an extended infomax algorithm for mixed subgaussian and supergaussian sources. *Neural Comput.* 11 417–441. 10.1162/089976699300016719 9950738

[B26] Leicht G. Andreou C. Polomac N. Lanig C. Schöttle D. Lambert M. (2015). Reduced auditory evoked gamma band response and cognitive processing deficits in first episode schizophrenia. *World J. Biol. Psychiatry* 16 387–397. 10.3109/15622975.2015.1017605 25774562

[B27] Leicht G. Kirsch V. Giegling I. Karch S. Hantschk I. Möller H.-J. (2010). Reduced early auditory evoked gamma-band response in patients with schizophrenia. *Biol. Psychiatry* 67 224–231. 10.1016/j.biopsych.2009.07.033 19765689

[B28] Lenz D. Fischer S. Schadow J. Bogerts B. Herrmann C. S. (2011). Altered evoked gamma-band responses as a neurophysiological marker of schizophrenia? *Int. J. Psychophysiol.* 79 25–31. 10.1016/j.ijpsycho.2010.08.002 20705107

[B29] Lisman J. (2012). Excitation, inhibition, local oscillations, or large-scale loops: what causes the symptoms of schizophrenia? *Curr. Opin. Neurobiol.* 22 537–544. 10.1016/j.conb.2011.10.018 22079494 PMC3302967

[B30] Maris E. (2012). Statistical testing in electrophysiological studies. *Psychophysiology* 49 549–565. 10.1111/j.1469-8986.2011.01320.x 22176204

[B31] Maris E. Oostenveld R. (2007). Nonparametric statistical testing of EEG- and MEG-data. *J. Neurosci. Methods* 164 177–190. 10.1016/j.jneumeth.2007.03.024 17517438

[B32] Martin B. Giersch A. Huron C. van Wassenhove V. (2013). Temporal event structure and timing in schizophrenia: preserved binding in a longer “now”. *Neuropsychologia* 51 358–371. 10.1016/j.neuropsychologia.2012.07.002 22813430

[B33] McGhie A. Chapman J. (1961). Disorders of attention and perception in early schizophrenia. *Br. J. Med. Psychol.* 34 103–116. 10.1111/j.2044-8341.1961.tb00936.x 13773940

[B34] Mishra J. Martinez A. Hillyard S. A. (2008). Cortical processes underlying sound-induced flash fusion. *Brain Res.* 1242 102–115. 10.1016/j.brainres.2008.05.023 18585695 PMC2584169

[B35] Mishra J. Martínez A. Hillyard S. A. (2010). Effect of attention on early cortical processes associated with the sound-induced extra flash illusion. *J. Cogn. Neurosci.* 22 1714–1729. 10.1162/jocn.2009.21295 19583464

[B36] Mishra J. Martinez A. Sejnowski T. J. Hillyard S. A. (2007). Early cross-modal interactions in auditory and visual cortex underlie a sound-induced visual illusion. *J. Neurosci.* 27 4120–4131. 10.1523/JNEUROSCI.4912-06.2007 17428990 PMC2905511

[B37] Norton D. Ongur D. Stromeyer C. III Chen Y. (2008). Altered “three-flash” illusion in response to two light pulses in schizophrenia. *Schizophr. Res.* 103 275–282. 10.1016/j.schres.2008.03.002 18423984 PMC2553681

[B38] Onitsuka T. Oribe N. Nakamura I. Kanba S. (2013). Review of neurophysiological findings in patients with schizophrenia. *Psychiatry Clin. Neurosci.* 67 461–470. 10.1111/pcn.12090 24102977

[B39] Oostenveld R. Fries P. Maris E. Schoffelen J.-M. (2011). FieldTrip: open source software for advanced analysis of MEG, EEG, and invasive electrophysiological data. *Comput. Intell. Neurosci.* 2011:156869 10.1155/2011/156869 PMC3021840 21253357

[B40] Pearl D. Yodashkin-Porat D. Katz N. Valevski A. Aizenberg D. Sigler M. (2009). Differences in audiovisual integration, as measured by McGurk phenomenon, among adult and adolescent patients with schizophrenia and age-matched healthy control groups. *Compr. Psychiatry* 50 186–192. 10.1016/j.comppsych.2008.06.004 19216897

[B41] Pittman-Polletta B. R. Kocsis B. Vijayan S. Whittington M. A. Kopell N. J. (2015). Brain rhythms connect impaired inhibition to altered cognition in schizophrenia. *Biol. Psychiatry* 77 1020–1030. 10.1016/j.biopsych.2015.02.005 25850619 PMC4444389

[B42] Rentrop M. Roth A. Rodewald K. Simon J. Metzler S. Walther S. (2011). Temporal variability and spatial diffusion of the N2 event-related potential in high-functioning patients with schizophrenia. *Schizophr. Res.* 131 206–213. 10.1016/j.schres.2011.06.020 21745725

[B43] Roa Romero Y. Keil J. Balz J. Niedeggen M. Gallinat J. Senkowski D. (2016a). Alpha-band oscillations reflect altered multisensory processing of the McGurk illusion in schizophrenia. *Front. Hum. Neurosci.* 10:41 10.3389/fnhum.2016.00041 PMC4751891 26903845

[B44] Roa Romero Y. Keil J. Balz J. Gallinat J. Senkowski D. (2016b). Reduced frontal theta oscillations indicate altered crossmodal prediction error processing in schizophrenia. *J. Neurophysiol.* 116 1396–1407. 10.1152/jn.00096.2016 27358314 PMC5040384

[B45] Ross L. A. Saint-Amour D. Leavitt V. M. Molholm S. Javitt D. C. Foxe J. J. (2007). Impaired multisensory processing in schizophrenia: deficits in the visual enhancement of speech comprehension under noisy environmental conditions. *Schizophr. Res.* 97 173–183. 10.1016/j.schres.2007.08.008 17928202

[B46] Senkowski D. Gallinat J. (2015). Dysfunctional prefrontal gamma-band oscillations reflect working memory and other cognitive deficits in schizophrenia. *Biol. Psychiatry* 77 1010–1019. 10.1016/j.biopsych.2015.02.034 25847179

[B47] Senkowski D. Saint-Amour D. Kelly S. P. Foxe J. J. (2007). Multisensory processing of naturalistic objects in motion: a high-density electrical mapping and source estimation study. *Neuroimage* 36 877–888. 10.1016/j.neuroimage.2007.01.053 17481922

[B48] Shakow D. (1963). Psychological deficit in schizophrenia. *Behav. Sci.* 8 275–305. 10.1002/bs.3830080402 4867203

[B49] Shams L. Kamitani Y. Shimojo S. (2000). Illusions. What you see is what you hear. *Nature* 408: 788 10.1038/35048669 11130706

[B50] Singer W. Gray C. M. (1995). Visual feature integration and the temporal correlation hypothesis. *Annu. Rev. Neurosci.* 18 555–586. 10.1146/annurev.ne.18.030195.003011 7605074

[B51] Spencer K. M. Salisbury D. F. Shenton M. E. Mccarley R. W. (2008). Gamma-band auditory steady-state responses are impaired in first episode psychosis. *Biol. Psychiatry* 64 369–375. 10.1016/j.biopsych.2008.02.021 18400208 PMC2579257

[B52] Stekelenburg J. J. Maes J. P. Van Gool A. R. Sitskoorn M. Vroomen J. (2013). Deficient multisensory integration in schizophrenia: an event-related potential study. *Schizophr. Res.* 147 253–261. 10.1016/j.schres.2013.04.038 23707640

[B53] Stevenson R. A. Park S. Cochran C. McIntosh L. G. Noel J. P. Barense M. D. (2016). The associations between multisensory temporal processing and symptoms of schizophrenia. *Schizophr. Res.* 10.1016/j.schres.2016.09.035 [Epub ahead of print]. PMC5463449 27746052

[B54] Stone D. B. Coffman B. A. Bustillo J. R. Aine C. J. Stephen J. M. (2014). Multisensory stimuli elicit altered oscillatory brain responses at gamma frequencies in patients with schizophrenia. *Front. Hum. Neurosci.* 8:788 10.3389/fnhum.2014.00788 PMC4220133 25414652

[B55] Stone D. B. Urrea L. J. Aine C. J. Bustillo J. R. Clark V. P. Stephen J. M. (2011). Unisensory processing and multisensory integration in schizophrenia: a high-density electrical mapping study. *Neuropsychologia* 49 3178–3187. 10.1016/j.neuropsychologia.2011.07.017 21807011 PMC3632320

[B56] Surguladze S. A. Calvert G. A. Brammer M. J. Campbell R. Bullmore E. T. Giampietro V. (2001). Audio-visual speech perception in schizophrenia: an fMRI study. *Psychiatry Res.* 106 1–14. 10.1016/S0925-4927(00)00081-0 11231095

[B57] Tseng H. Bossong M. G. Modinos G. Chen K. McGuire P. Allen P. (2015). A systematic review of multisensory cognitive-affective integration in schizophrenia. *Neurosci. Biobehav. Rev.* 55 444–452. 10.1016/j.neubiorev.2015.04.019 25956248

[B58] Uhlhaas P. J. Linden D. E. J. Singer W. Haenschel C. Lindner M. Maurer K. (2006). Dysfunctional long-range coordination of neural activity during Gestalt perception in schizophrenia. *J. Neurosci.* 26 8168–8175. 10.1523/JNEUROSCI.2002-06.2006 16885230 PMC6673788

[B59] Uhlhaas P. J. Singer W. (2015). Oscillations and neuronal dynamics in schizophrenia: the search for basic symptoms and translational opportunities. *Biol. Psychiatry* 77 1001–1009. 10.1016/j.biopsych.2014.11.019 25676489

[B60] van Wassenhove V. Grant K. W. Poeppel D. (2005). Visual speech speeds up the neural processing of auditory speech. *Proc. Natl. Acad. Sci. U.S.A.* 102 1181–1186. 10.1073/pnas.0408949102 15647358 PMC545853

[B61] Vlcek P. Bob P. Raboch J. (2014). Sensory disturbances, inhibitory deficits, and the P50 wave in schizophrenia. *Neuropsychiatr. Dis. Treat.* 10 1309–1315. 10.2147/NDT.S64219 25075189 PMC4106969

[B62] Wynn J. K. Jahshan C. Green M. F. (2014). Multisensory integration in schizophrenia: a behavioural and event-related potential study. *Cogn. Neuropsychiatry* 19 319–336. 10.1080/13546805.2013.866892 24397788 PMC4103881

