# Supplementary material for: Beta/Gamma Oscillations and Event-Related Potentials Indicate Aberrant Multisensory Processing in Schizophrenia
Source: Front Psychol. 2016 Dec 6;7:1896. doi: 10.3389/fpsyg.2016.01896 (PMC5138197; doi:10.3389/fpsyg.2016.01896)
Supplement: Supplementary file 1 [file Image_1.PDF]

Supplementary Material

Supplementary Figure 1

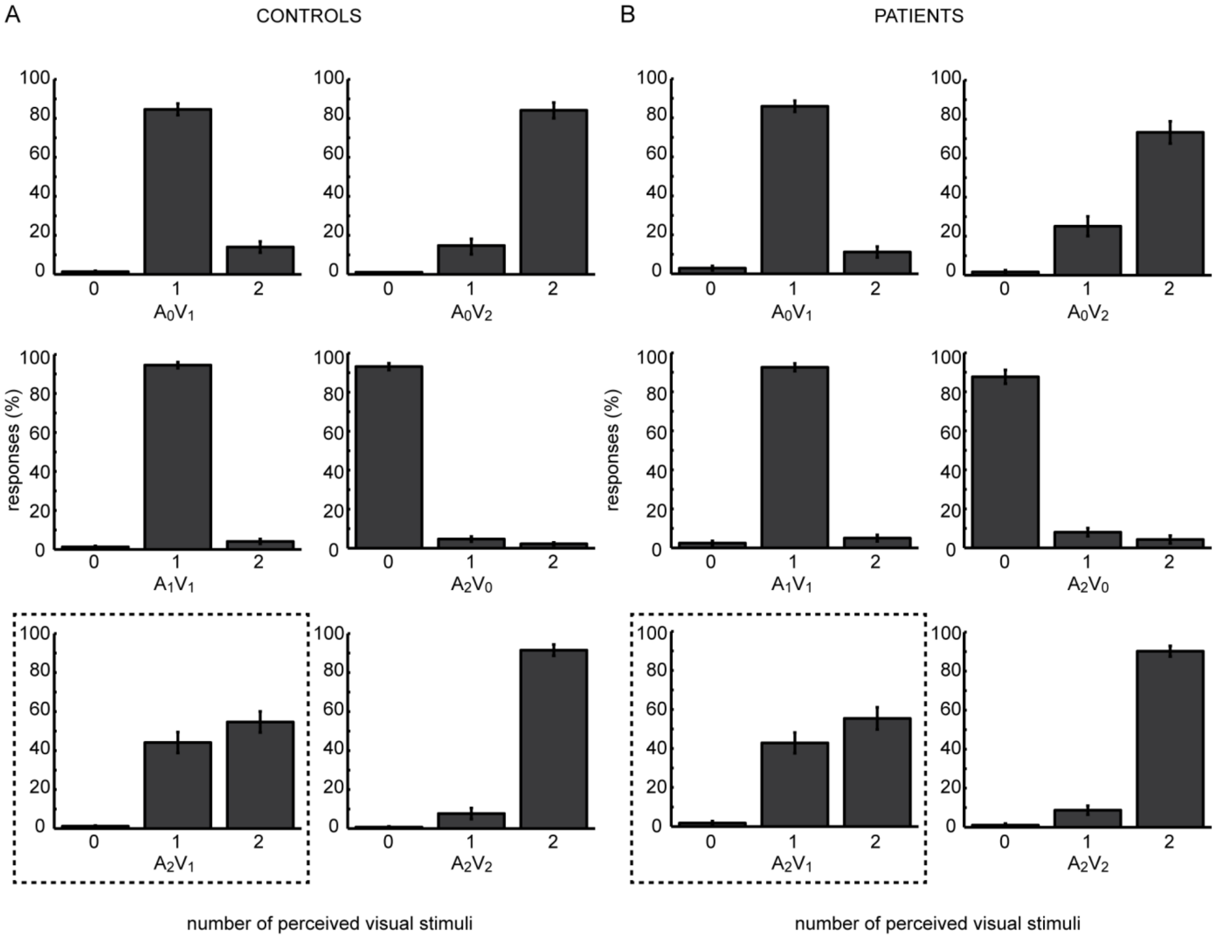

Supplementary Figure 1. Behavioral data of controls (A) and patients (B) for the six stimulus types in the sound-induced flash illusion paradigm. The indexed numbers denote the number of auditory (A) and visual (V) inputs in the six different stimulus types (e.g., two auditory inputs were presented together with two visual inputs in A_2V_2 trials). The gray bars denote the numbers of perceived visual stimuli (with SEM). The critical A_2V_1 trials are highlighted in a box.

Supplementary Figure 2

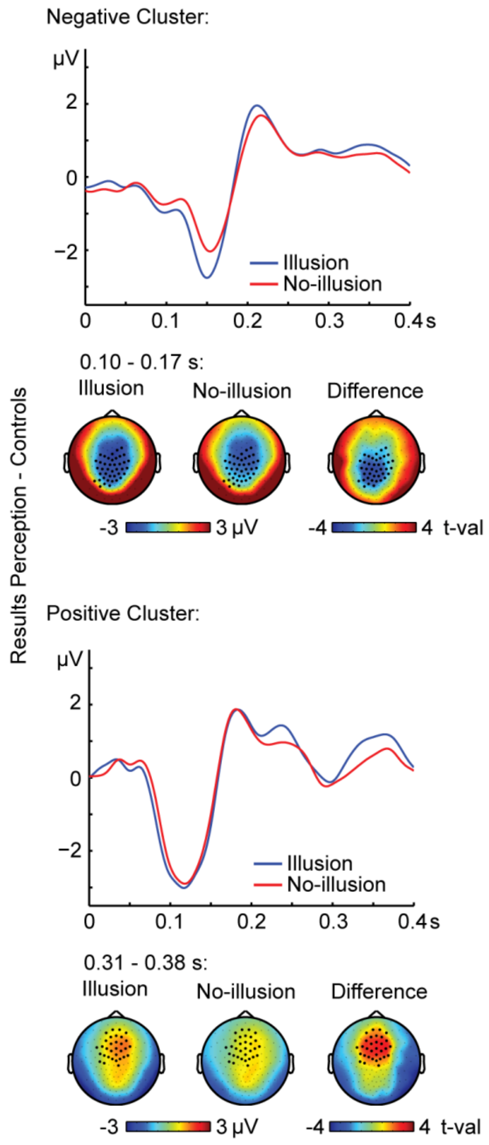

Supplementary Figure 2. ERP effect of multisensory A_2V_1 trials of healthy controls. Outcome of the non-parametric tests with cluster-based correction for multiple comparisons between Perceptions (illusion vs. no-illusion). The negative and the positive cluster are illustrated separately. The upper planes illustrate the ERP results for Perception. The lower planes depict topographic maps for the observed results with highlighted significant cluster electrodes. Time-point 0 indicates the onset of the first visual stimulus.

Supplementary Figure 3

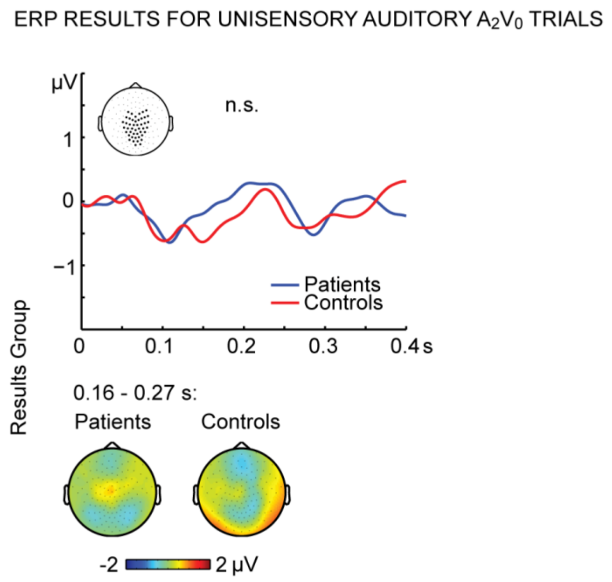

Supplementary Figure 3. ERP results of unisensory auditory A_2V_0 trials. Outcome of the non-parametric tests with cluster-based correction for multiple comparisons between Groups (SCZ vs. HC). The upper plane illustrates the ERP results for Group. The lower plane depicts topographic maps for the observed results. Time-point 0 indicates the onset of the first visual stimulus.

Supplementary Figure 4

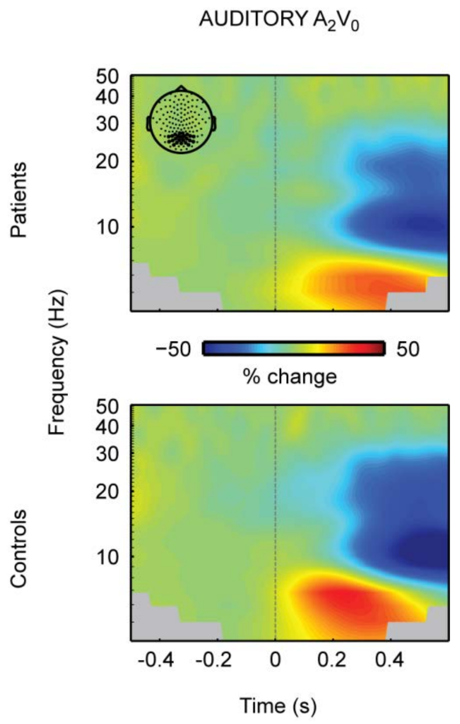

Supplementary Figure 4. Total oscillatory power over occipital cortex of unisensory auditory A_2V_0 trials. Time-frequency representations at occipital electrodes in response to unisensory auditory trials. There were no significant differences between SCZ and HC in 25-35 Hz total power in auditory A_2V_0 trials. Time-point 0 indicates the onset of the first auditory and visual stimulus.
